# 2,2′-Bis(ferrocenylmethyl)-5,5′-(*m*-phenyl­ene)di-2*H*-tetra­zole

**DOI:** 10.1107/S1600536809022442

**Published:** 2009-06-17

**Authors:** Fang Chen

**Affiliations:** a Ordered Matter Science Research Center, College of Chemistry and Chemical Engineering, Southeast University, Nanjing 210096, People’s Republic of China

## Abstract

In the title compound, [Fe_2_(C_5_H_5_)_2_(C_20_H_16_N_8_)], one of the unsubstituted cyclo­penta­diene (Cp) rings is disordered over two positions, with site-occupancy factors of 0.609 (19) and 0.391 (19). The dihedral angle formed by the benzene ring with the tetra­zole rings are 51.86 (15) and 3.76 (11)°. In the crystal structure, centrosymmetrically related mol­ecules are linked into dimers by inter­molecular C—H⋯N hydrogen-bonding inter­actions.

## Related literature

For the applications of ferrocene derivatives, see: Yang *et al.* (2002 ▶); Togni & Hayashi (1995 ▶); Long (1995 ▶); Roberto *et al.* (2000 ▶). For the crystal structures of related compounds, see: Hess *et al.* (1999 ▶); Base *et al.* (2002 ▶); Cao & Ye (2008 ▶).
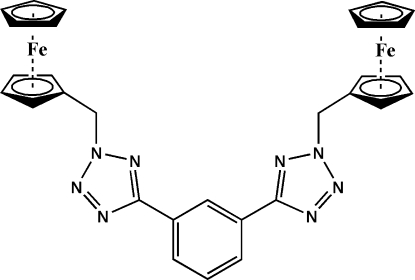


## Experimental

### 

#### Crystal data

[Fe_2_(C_5_H_5_)_2_(C_20_H_16_N_8_)]
*M* *_r_* = 610.29
Triclinic, 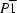

*a* = 10.9665 (3) Å
*b* = 11.0860 (2) Å
*c* = 12.9410 (3) Å
α = 74.982 (4)°
β = 67.793 (4)°
γ = 70.738 (5)°
*V* = 1358.69 (8) Å^3^
*Z* = 2
Mo *K*α radiation
μ = 1.10 mm^−1^
*T* = 293 K
0.25 × 0.15 × 0.10 mm

#### Data collection

Rigaku SCXmini diffractometer
Absorption correction: none
13984 measured reflections
6158 independent reflections
3375 reflections with *I* > 2σ(*I*)
*R*_int_ = 0.071

#### Refinement

*R*[*F* ^2^ > 2σ(*F* ^2^)] = 0.056
*wR*(*F* ^2^) = 0.117
*S* = 0.95
6158 reflections
407 parameters
621 restraints
Δρ_max_ = 0.34 e Å^−3^
Δρ_min_ = −0.40 e Å^−3^

### 

Data collection: *CrystalClear* (Rigaku, 2005 ▶); cell refinement: *CrystalClear*; data reduction: *CrystalClear*; program(s) used to solve structure: *SHELXS97* (Sheldrick, 2008 ▶); program(s) used to refine structure: *SHELXL97* (Sheldrick, 2008 ▶); molecular graphics: *SHELXTL* (Sheldrick, 2008 ▶); software used to prepare material for publication: *SHELXL97*.

## Supplementary Material

Crystal structure: contains datablocks I, global. DOI: 10.1107/S1600536809022442/rz2333sup1.cif

Structure factors: contains datablocks I. DOI: 10.1107/S1600536809022442/rz2333Isup2.hkl

Additional supplementary materials:  crystallographic information; 3D view; checkCIF report

## Figures and Tables

**Table 1:** Hydrogen-bond geometry (Å, °)

*D*—H⋯*A*	*D*—H	H⋯*A*	*D*⋯*A*	*D*—H⋯*A*
C20—H20*B*⋯N2^i^	0.97	2.49	3.391 (5)	154

Symmetry code: (i) 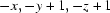.

**Fig. 1.**
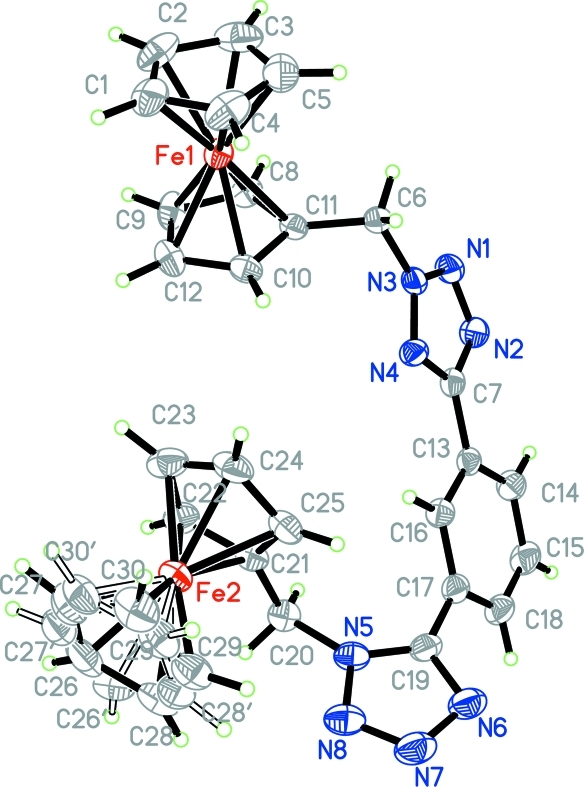
The molecular structure of the title compound, with displacement ellipsoids drawn at the 30% probability level.

## supplementary crystallographic information

### Comment

The chemistry of ferrocene has received much attention because of its applications in many fields, such as catalysis (Yang *et al.*, 2002), organic or organometallic synthesis and materials (Togni & Hayashi, 1995), and non-linear optical (NLO) materials (Long, 1995; Roberto *et al.*, 2000). As part of our continuing studies on new ferrocene compounds, the crystal structure of the title compound is reported herein.

In the title compound (Fig. 1), one of the unsubstituted cyclopentadiene (Cp) rings is disordered over two positions, with site-occupancy factors of 0.609 (19) and 0.391 (19) for the major and minor components, respectively. The dihedral angles formed within the (Cp)_2_Fe unit by the disordered components with the substituted Cp ring are 1.1 (4) and 1.7 (6)°. The benzene ring forms dihedral angles of 3.76 (11) and 51.86 (15)° with the N1–N4/C7 and N5–N8/C19 tetrazole rings, respectively. The Fe—C distances range from 2.00 (2) to 2.06 (3) Å, and are in agreement with those reported for related compounds (Hess *et al.*, 1999; Base *et al.*, 2002). In the crystal structure, centrosymmetrically related molecules are linked into dimers by intermolecular C—H···N hydrogen bonding interactions (Table 1).

### Experimental

To a mixture of [Fe(C_5_H_5_)(C_5_H_4_)N^+^(CH_3_)_3_I^-^] (10 mmol) in H_2_O (50 ml) was added 5-(3-(2*H*-tetrazol-5-yl)phenyl)-2*H*-tetrazole (5 mmol) and the mixture was heated to reflux temperature for 5 h. Then, the formed yellow precipitate was filtered. Crystals suitable for X-ray diffraction analysis were obtained by slow evaporation of a dichloromethane solution at room temperature after 3 days.

### Refinement

Positional parameters of all the H atoms were calculated geometrically and were allowed to ride on their parent atoms, with C—H = 0.93-0.98 Å and with *U*_iso_(H) = 1.2*U*_iso_(C). One cyclopentadiene rings is disordered over two positions, with refined site-occupancy factors of 0.609 (19) and 0.391 (19) for the major and minor components, respectively. Soft proximity (SIMU) and rigid-bond restraints (DELU) were applied to the anisotropic displacement parameters.

### Crystal data

**Table:**

[Fe_2_(C_5_H_5_)_2_(C_20_H_16_N_8_)]	*Z* = 2
*M**_r_* = 610.29	*F*(000) = 628
Triclinic, *P*1	*D*_x_ = 1.492 Mg m^−^^3^
Hall symbol: -P 1	Mo *K*α radiation, λ = 0.71073 Å
*a* = 10.9665 (3) Å	Cell parameters from 2765 reflections
*b* = 11.0860 (2) Å	θ = 2.8–27.5°
*c* = 12.9410 (3) Å	µ = 1.10 mm^−^^1^
α = 74.982 (4)°	*T* = 293 K
β = 67.793 (4)°	Prism, colorless
γ = 70.738 (5)°	0.25 × 0.15 × 0.10 mm
*V* = 1358.69 (8) Å^3^	

### Data collection

**Table:**

Rigaku SCXmini diffractometer	3375 reflections with *I* > 2σ(*I*)
Radiation source: fine-focus sealed tube	*R*_int_ = 0.071
graphite	θ_max_ = 27.5°, θ_min_ = 2.8°
Detector resolution: 13.6612 pixels mm^-1^	*h* = −14→14
ω scans	*k* = −14→14
13984 measured reflections	*l* = −16→16
6158 independent reflections	

### Refinement

**Table:**

Refinement on *F*^2^	Primary atom site location: structure-invariant direct methods
Least-squares matrix: full	Secondary atom site location: difference Fourier map
*R*[*F*^2^ > 2σ(*F*^2^)] = 0.056	Hydrogen site location: inferred from neighbouring sites
*wR*(*F*^2^) = 0.117	*w* = 1/[σ^2^(*F*_o_^2^) + (0.0405*P*)^2^] where *P* = (*F*_o_^2^ + 2*F*_c_^2^)/3
*S* = 0.95	(Δ/σ)_max_ < 0.001
6158 reflections	Δρ_max_ = 0.34 e Å^−^^3^
407 parameters	Δρ_min_ = −0.40 e Å^−^^3^
621 restraints	

### Special details

**Table:**

Geometry. All e.s.d.'s (except the e.s.d. in the dihedral angle between two l.s. planes) are estimated using the full covariance matrix. The cell e.s.d.'s are taken into account individually in the estimation of e.s.d.'s in distances, angles and torsion angles; correlations between e.s.d.'s in cell parameters are only used when they are defined by crystal symmetry. An approximate (isotropic) treatment of cell e.s.d.'s is used for estimating e.s.d.'s involving l.s. planes.
Refinement. Refinement of *F*^2^ against ALL reflections. The weighted *R*-factor *wR* and goodness of fit *S* are based on *F*^2^, conventional *R*-factors *R* are based on *F*, with *F* set to zero for negative *F*^2^. The threshold expression of *F*^2^ > σ(*F*^2^) is used only for calculating *R*-factors(gt) *etc*. and is not relevant to the choice of reflections for refinement. *R*-factors based on *F*^2^ are statistically about twice as large as those based on *F*, and *R*- factors based on ALL data will be even larger.

### Fractional atomic coordinates and isotropic or equivalent isotropic displacement parameters (Å^2^)

**Table:**

	*x*	*y*	*z*	*U*_iso_*/*U*_eq_	Occ. (<1)
Fe1	0.58316 (5)	0.73009 (5)	0.09634 (4)	0.04250 (16)	
Fe2	−0.12863 (5)	1.12049 (5)	0.23589 (5)	0.04961 (17)	
N1	0.3674 (3)	0.4387 (3)	0.4787 (3)	0.0521 (8)	
N2	0.2452 (3)	0.4283 (3)	0.5485 (2)	0.0511 (8)	
N3	0.3506 (3)	0.5609 (3)	0.4298 (2)	0.0433 (7)	
N4	0.2231 (3)	0.6324 (3)	0.4627 (2)	0.0449 (7)	
N5	−0.3040 (3)	0.9265 (3)	0.5149 (3)	0.0505 (8)	
N6	−0.3789 (3)	0.9349 (4)	0.6942 (3)	0.0678 (10)	
N7	−0.4382 (4)	1.0472 (4)	0.6374 (3)	0.0740 (11)	
N8	−0.3940 (3)	1.0439 (3)	0.5299 (3)	0.0641 (9)	
C1	0.6792 (4)	0.8657 (4)	−0.0129 (4)	0.0667 (11)	
H1B	0.6448	0.9332	−0.0685	0.080*	
C2	0.7600 (5)	0.7410 (5)	−0.0309 (4)	0.0745 (13)	
H2B	0.7934	0.7059	−0.1016	0.089*	
C3	0.7860 (4)	0.6750 (4)	0.0710 (5)	0.0806 (14)	
H3A	0.8415	0.5864	0.0837	0.097*	
C4	0.6553 (4)	0.8767 (4)	0.0987 (4)	0.0712 (12)	
H4B	0.6010	0.9536	0.1348	0.085*	
C5	0.7204 (5)	0.7613 (5)	0.1493 (4)	0.0784 (13)	
H5A	0.7206	0.7426	0.2275	0.094*	
C6	0.4641 (3)	0.6131 (4)	0.3473 (3)	0.0461 (9)	
H6A	0.5497	0.5488	0.3455	0.055*	
H6B	0.4651	0.6887	0.3712	0.055*	
C7	0.1593 (4)	0.5462 (3)	0.5371 (3)	0.0414 (8)	
C8	0.5128 (4)	0.5690 (3)	0.1457 (3)	0.0471 (9)	
H8A	0.5679	0.4793	0.1525	0.057*	
C9	0.4797 (4)	0.6434 (4)	0.0480 (3)	0.0557 (10)	
H9A	0.5087	0.6143	−0.0250	0.067*	
C10	0.3811 (3)	0.7707 (3)	0.1868 (3)	0.0471 (9)	
H10A	0.3297	0.8451	0.2268	0.057*	
C11	0.4521 (3)	0.6492 (3)	0.2320 (3)	0.0389 (8)	
C12	0.3984 (4)	0.7668 (4)	0.0744 (3)	0.0557 (10)	
H12A	0.3619	0.8385	0.0224	0.067*	
C13	0.0124 (3)	0.5768 (3)	0.6002 (3)	0.0403 (8)	
C14	−0.0457 (4)	0.4852 (4)	0.6813 (3)	0.0531 (10)	
H14A	0.0087	0.4030	0.6970	0.064*	
C15	−0.1848 (4)	0.5145 (4)	0.7397 (3)	0.0603 (11)	
H15A	−0.2236	0.4515	0.7925	0.072*	
C16	−0.0695 (3)	0.6998 (3)	0.5781 (3)	0.0453 (9)	
H16A	−0.0313	0.7619	0.5234	0.054*	
C17	−0.2083 (4)	0.7304 (4)	0.6371 (3)	0.0476 (9)	
C18	−0.2648 (4)	0.6376 (4)	0.7188 (3)	0.0576 (10)	
H18A	−0.3572	0.6584	0.7598	0.069*	
C19	−0.2952 (4)	0.8605 (4)	0.6160 (3)	0.0507 (9)	
C20	−0.2429 (4)	0.8867 (3)	0.4030 (3)	0.0512 (9)	
H20A	−0.3118	0.9147	0.3658	0.061*	
H20B	−0.2127	0.7931	0.4117	0.061*	
C21	−0.1239 (4)	0.9415 (3)	0.3297 (3)	0.0479 (9)	
C22	−0.0586 (4)	0.9316 (4)	0.2131 (3)	0.0570 (10)	
H22A	−0.0855	0.8918	0.1685	0.068*	
C23	0.0511 (4)	0.9912 (4)	0.1719 (4)	0.0677 (11)	
H23A	0.1136	0.9992	0.0942	0.081*	
C24	0.0544 (4)	1.0356 (4)	0.2636 (4)	0.0656 (11)	
H24A	0.1192	1.0809	0.2603	0.079*	
C25	−0.0539 (4)	1.0055 (4)	0.3609 (4)	0.0571 (10)	
H25A	−0.0765	1.0260	0.4363	0.069*	
C26	−0.262 (2)	1.2163 (15)	0.1517 (16)	0.080 (4)	0.609 (19)
H26A	−0.2866	1.1784	0.1045	0.096*	0.609 (19)
C27	−0.153 (2)	1.2725 (19)	0.1138 (17)	0.085 (5)	0.609 (19)
H27A	−0.0906	1.2802	0.0362	0.102*	0.609 (19)
C28	−0.3298 (12)	1.2208 (12)	0.2681 (16)	0.064 (3)	0.609 (19)
H28A	−0.4097	1.1886	0.3160	0.077*	0.609 (19)
C29	−0.2581 (15)	1.2829 (11)	0.3010 (12)	0.064 (3)	0.609 (19)
H29A	−0.2792	1.2980	0.3780	0.076*	0.609 (19)
C30	−0.1475 (16)	1.3173 (12)	0.2046 (17)	0.075 (4)	0.609 (19)
H30A	−0.0822	1.3612	0.2025	0.090*	0.609 (19)
C30'	−0.132 (3)	1.292 (3)	0.126 (3)	0.063 (4)	0.391 (19)
H30B	−0.0530	1.3223	0.0719	0.076*	0.391 (19)
C28'	−0.304 (2)	1.256 (2)	0.2895 (18)	0.069 (6)	0.391 (19)
H28B	−0.3656	1.2559	0.3675	0.082*	0.391 (19)
C29'	−0.196 (3)	1.310 (2)	0.244 (3)	0.070 (5)	0.391 (19)
H29B	−0.1686	1.3552	0.2838	0.084*	0.391 (19)
C27'	−0.207 (2)	1.229 (2)	0.1052 (17)	0.065 (4)	0.391 (19)
H27B	−0.1850	1.2002	0.0333	0.079*	0.391 (19)
C26'	−0.3154 (16)	1.2047 (19)	0.204 (3)	0.065 (5)	0.391 (19)
H26B	−0.3835	1.1608	0.2119	0.077*	0.391 (19)

### Atomic displacement parameters (Å^2^)

**Table:**

	*U*^11^	*U*^22^	*U*^33^	*U*^12^	*U*^13^	*U*^23^
Fe1	0.0497 (3)	0.0375 (3)	0.0420 (3)	−0.0182 (2)	−0.0125 (3)	−0.0033 (2)
Fe2	0.0487 (3)	0.0428 (3)	0.0586 (4)	−0.0086 (3)	−0.0235 (3)	−0.0045 (3)
N1	0.0493 (19)	0.0487 (18)	0.051 (2)	−0.0080 (15)	−0.0195 (16)	0.0035 (15)
N2	0.053 (2)	0.0471 (18)	0.049 (2)	−0.0168 (16)	−0.0151 (16)	0.0017 (15)
N3	0.0410 (17)	0.0488 (18)	0.0394 (17)	−0.0135 (15)	−0.0171 (14)	0.0032 (14)
N4	0.0400 (17)	0.0440 (17)	0.0421 (18)	−0.0092 (14)	−0.0087 (14)	−0.0017 (14)
N5	0.0438 (18)	0.0413 (17)	0.066 (2)	−0.0079 (15)	−0.0153 (16)	−0.0151 (16)
N6	0.052 (2)	0.075 (2)	0.070 (2)	−0.0050 (19)	−0.0104 (19)	−0.031 (2)
N7	0.058 (2)	0.078 (3)	0.082 (3)	−0.004 (2)	−0.012 (2)	−0.038 (2)
N8	0.052 (2)	0.053 (2)	0.084 (3)	−0.0044 (17)	−0.018 (2)	−0.0223 (19)
C1	0.071 (3)	0.061 (2)	0.064 (3)	−0.037 (2)	−0.012 (2)	0.009 (2)
C2	0.075 (3)	0.083 (3)	0.062 (3)	−0.046 (3)	0.012 (2)	−0.024 (2)
C3	0.047 (2)	0.057 (3)	0.119 (4)	−0.015 (2)	−0.017 (3)	0.005 (2)
C4	0.075 (3)	0.065 (3)	0.077 (3)	−0.041 (2)	−0.001 (2)	−0.022 (2)
C5	0.076 (3)	0.115 (4)	0.065 (3)	−0.063 (3)	−0.026 (2)	0.007 (3)
C6	0.037 (2)	0.057 (2)	0.043 (2)	−0.0168 (18)	−0.0117 (17)	−0.0018 (17)
C7	0.047 (2)	0.046 (2)	0.036 (2)	−0.0186 (17)	−0.0144 (16)	−0.0042 (16)
C8	0.052 (2)	0.0333 (17)	0.053 (2)	−0.0173 (16)	−0.0106 (18)	−0.0036 (16)
C9	0.084 (3)	0.056 (2)	0.042 (2)	−0.037 (2)	−0.022 (2)	−0.0051 (17)
C10	0.0419 (19)	0.0390 (19)	0.060 (2)	−0.0052 (16)	−0.0199 (18)	−0.0081 (17)
C11	0.0354 (19)	0.0381 (18)	0.0444 (19)	−0.0103 (15)	−0.0154 (15)	−0.0033 (15)
C12	0.071 (3)	0.047 (2)	0.063 (3)	−0.021 (2)	−0.039 (2)	0.0049 (19)
C13	0.0424 (19)	0.049 (2)	0.035 (2)	−0.0189 (16)	−0.0132 (16)	−0.0040 (16)
C14	0.054 (2)	0.054 (2)	0.050 (2)	−0.0217 (19)	−0.0147 (19)	0.0017 (18)
C15	0.061 (3)	0.068 (3)	0.047 (2)	−0.035 (2)	−0.007 (2)	0.006 (2)
C16	0.045 (2)	0.051 (2)	0.044 (2)	−0.0214 (17)	−0.0119 (17)	−0.0059 (17)
C17	0.045 (2)	0.060 (2)	0.043 (2)	−0.0163 (18)	−0.0133 (18)	−0.0145 (18)
C18	0.049 (2)	0.078 (3)	0.044 (2)	−0.027 (2)	−0.0056 (19)	−0.007 (2)
C19	0.040 (2)	0.060 (2)	0.049 (2)	−0.0130 (18)	−0.0058 (19)	−0.0161 (19)
C20	0.054 (2)	0.046 (2)	0.058 (2)	−0.0131 (18)	−0.0181 (19)	−0.0133 (18)
C21	0.041 (2)	0.0422 (19)	0.059 (2)	−0.0066 (16)	−0.0187 (17)	−0.0065 (17)
C22	0.054 (2)	0.048 (2)	0.064 (3)	−0.0078 (18)	−0.014 (2)	−0.0160 (19)
C23	0.049 (2)	0.049 (2)	0.080 (3)	−0.0033 (17)	−0.005 (2)	−0.004 (2)
C24	0.043 (2)	0.062 (3)	0.090 (3)	−0.0196 (19)	−0.030 (2)	0.011 (2)
C25	0.053 (2)	0.056 (2)	0.068 (2)	−0.0150 (19)	−0.034 (2)	0.0041 (19)
C26	0.082 (10)	0.079 (7)	0.087 (9)	0.009 (7)	−0.060 (8)	−0.015 (8)
C27	0.094 (9)	0.061 (8)	0.078 (5)	−0.001 (6)	−0.034 (6)	0.011 (5)
C28	0.052 (4)	0.052 (6)	0.096 (9)	−0.003 (3)	−0.044 (5)	−0.009 (5)
C29	0.067 (7)	0.047 (5)	0.085 (6)	−0.002 (4)	−0.038 (5)	−0.020 (4)
C30	0.086 (8)	0.038 (4)	0.102 (11)	−0.024 (5)	−0.040 (7)	0.013 (6)
C30'	0.066 (8)	0.053 (6)	0.078 (8)	−0.021 (5)	−0.041 (6)	0.013 (6)
C28'	0.065 (8)	0.052 (9)	0.068 (8)	0.009 (6)	−0.020 (6)	−0.007 (6)
C29'	0.084 (12)	0.045 (4)	0.089 (11)	−0.004 (6)	−0.047 (9)	−0.012 (7)
C27'	0.061 (11)	0.076 (9)	0.064 (8)	−0.017 (8)	−0.031 (6)	−0.002 (7)
C26'	0.039 (7)	0.061 (8)	0.086 (14)	−0.002 (6)	−0.018 (8)	−0.015 (9)

### Geometric parameters (Å, °)

**Table:**

Fe1—C11	2.020 (3)	C8—H8A	0.9800
Fe1—C3	2.021 (4)	C9—C12	1.411 (5)
Fe1—C5	2.025 (4)	C9—H9A	0.9800
Fe1—C2	2.031 (4)	C10—C12	1.404 (5)
Fe1—C8	2.034 (3)	C10—C11	1.417 (4)
Fe1—C10	2.034 (3)	C10—H10A	0.9800
Fe1—C4	2.040 (4)	C12—H12A	0.9800
Fe1—C9	2.042 (4)	C13—C14	1.381 (4)
Fe1—C12	2.045 (4)	C13—C16	1.390 (5)
Fe1—C1	2.046 (4)	C14—C15	1.389 (5)
Fe2—C29'	2.00 (2)	C14—H14A	0.9300
Fe2—C26	2.005 (13)	C15—C18	1.379 (5)
Fe2—C27	2.013 (19)	C15—H15A	0.9300
Fe2—C28'	2.016 (19)	C16—C17	1.389 (4)
Fe2—C21	2.036 (4)	C16—H16A	0.9300
Fe2—C23	2.036 (4)	C17—C18	1.386 (5)
Fe2—C22	2.037 (4)	C17—C19	1.467 (5)
Fe2—C25	2.040 (4)	C18—H18A	0.9300
Fe2—C29	2.040 (10)	C20—C21	1.504 (5)
Fe2—C24	2.041 (4)	C20—H20A	0.9700
Fe2—C28	2.050 (11)	C20—H20B	0.9700
Fe2—C30'	2.06 (3)	C21—C25	1.413 (5)
N1—N3	1.322 (4)	C21—C22	1.421 (5)
N1—N2	1.326 (4)	C22—C23	1.424 (5)
N2—C7	1.346 (4)	C22—H22A	0.9800
N3—N4	1.326 (4)	C23—C24	1.414 (6)
N3—C6	1.475 (4)	C23—H23A	0.9800
N4—C7	1.336 (4)	C24—C25	1.422 (5)
N5—C19	1.345 (4)	C24—H24A	0.9800
N5—N8	1.358 (4)	C25—H25A	0.9800
N5—C20	1.464 (4)	C26—C27	1.39 (2)
N6—C19	1.333 (4)	C26—C28	1.413 (16)
N6—N7	1.366 (5)	C26—H26A	0.9800
N7—N8	1.294 (4)	C27—C30	1.42 (2)
C1—C4	1.397 (6)	C27—H27A	0.9800
C1—C2	1.397 (6)	C28—C29	1.427 (12)
C1—H1B	0.9800	C28—H28A	0.9800
C2—C3	1.415 (6)	C29—C30	1.445 (13)
C2—H2B	0.9800	C29—H29A	0.9800
C3—C5	1.399 (6)	C30—H30A	0.9800
C3—H3A	0.9800	C30'—C27'	1.38 (2)
C4—C5	1.376 (6)	C30'—C29'	1.46 (3)
C4—H4B	0.9800	C30'—H30B	0.9800
C5—H5A	0.9800	C28'—C29'	1.36 (3)
C6—C11	1.486 (4)	C28'—C26'	1.44 (2)
C6—H6A	0.9700	C28'—H28B	0.9800
C6—H6B	0.9700	C29'—H29B	0.9800
C7—C13	1.472 (4)	C27'—C26'	1.41 (2)
C8—C9	1.421 (5)	C27'—H27B	0.9800
C8—C11	1.428 (5)	C26'—H26B	0.9800
			
C11—Fe1—C3	121.64 (17)	C11—C6—H6A	109.2
C11—Fe1—C5	107.88 (16)	N3—C6—H6B	109.2
C3—Fe1—C5	40.47 (18)	C11—C6—H6B	109.2
C11—Fe1—C2	157.84 (17)	H6A—C6—H6B	107.9
C3—Fe1—C2	40.87 (18)	N4—C7—N2	112.2 (3)
C5—Fe1—C2	67.80 (19)	N4—C7—C13	124.0 (3)
C11—Fe1—C8	41.26 (13)	N2—C7—C13	123.8 (3)
C3—Fe1—C8	108.53 (17)	C9—C8—C11	107.6 (3)
C5—Fe1—C8	125.84 (18)	C9—C8—Fe1	69.9 (2)
C2—Fe1—C8	122.32 (16)	C11—C8—Fe1	68.85 (19)
C11—Fe1—C10	40.91 (13)	C9—C8—H8A	126.2
C3—Fe1—C10	156.7 (2)	C11—C8—H8A	126.2
C5—Fe1—C10	121.13 (18)	Fe1—C8—H8A	126.2
C2—Fe1—C10	160.30 (18)	C12—C9—C8	107.9 (3)
C8—Fe1—C10	68.72 (14)	C12—C9—Fe1	69.9 (2)
C11—Fe1—C4	124.03 (16)	C8—C9—Fe1	69.3 (2)
C3—Fe1—C4	67.56 (19)	C12—C9—H9A	126.0
C5—Fe1—C4	39.56 (17)	C8—C9—H9A	126.0
C2—Fe1—C4	67.42 (17)	Fe1—C9—H9A	126.0
C8—Fe1—C4	161.55 (17)	C12—C10—C11	108.3 (3)
C10—Fe1—C4	107.32 (16)	C12—C10—Fe1	70.3 (2)
C11—Fe1—C9	68.95 (14)	C11—C10—Fe1	69.01 (19)
C3—Fe1—C9	125.8 (2)	C12—C10—H10A	125.8
C5—Fe1—C9	162.9 (2)	C11—C10—H10A	125.8
C2—Fe1—C9	108.41 (17)	Fe1—C10—H10A	125.8
C8—Fe1—C9	40.83 (14)	C10—C11—C8	107.6 (3)
C10—Fe1—C9	68.23 (15)	C10—C11—C6	126.1 (3)
C4—Fe1—C9	156.12 (18)	C8—C11—C6	126.3 (3)
C11—Fe1—C12	68.45 (14)	C10—C11—Fe1	70.1 (2)
C3—Fe1—C12	162.1 (2)	C8—C11—Fe1	69.89 (19)
C5—Fe1—C12	155.6 (2)	C6—C11—Fe1	124.2 (2)
C2—Fe1—C12	124.69 (19)	C10—C12—C9	108.6 (3)
C8—Fe1—C12	68.31 (15)	C10—C12—Fe1	69.5 (2)
C10—Fe1—C12	40.26 (14)	C9—C12—Fe1	69.7 (2)
C4—Fe1—C12	121.09 (18)	C10—C12—H12A	125.7
C9—Fe1—C12	40.39 (14)	C9—C12—H12A	125.7
C11—Fe1—C1	160.25 (16)	Fe1—C12—H12A	125.7
C3—Fe1—C1	67.90 (18)	C14—C13—C16	119.3 (3)
C5—Fe1—C1	67.10 (18)	C14—C13—C7	121.1 (3)
C2—Fe1—C1	40.09 (16)	C16—C13—C7	119.6 (3)
C8—Fe1—C1	157.14 (16)	C13—C14—C15	120.7 (4)
C10—Fe1—C1	123.77 (16)	C13—C14—H14A	119.6
C4—Fe1—C1	39.99 (16)	C15—C14—H14A	119.6
C9—Fe1—C1	121.56 (17)	C18—C15—C14	119.6 (4)
C12—Fe1—C1	107.73 (17)	C18—C15—H15A	120.2
C29'—Fe2—C26	66.4 (9)	C14—C15—H15A	120.2
C29'—Fe2—C27	50.0 (10)	C17—C16—C13	120.4 (3)
C26—Fe2—C27	40.4 (6)	C17—C16—H16A	119.8
C29'—Fe2—C28'	39.6 (7)	C13—C16—H16A	119.8
C26—Fe2—C28'	53.2 (7)	C18—C17—C16	119.6 (4)
C27—Fe2—C28'	68.0 (9)	C18—C17—C19	119.6 (3)
C29'—Fe2—C21	143.7 (9)	C16—C17—C19	120.8 (3)
C26—Fe2—C21	124.8 (6)	C15—C18—C17	120.4 (4)
C27—Fe2—C21	161.4 (7)	C15—C18—H18A	119.8
C28'—Fe2—C21	114.6 (7)	C17—C18—H18A	119.8
C29'—Fe2—C23	138.0 (9)	N6—C19—N5	107.9 (4)
C26—Fe2—C23	124.2 (5)	N6—C19—C17	125.7 (4)
C27—Fe2—C23	108.3 (7)	N5—C19—C17	126.4 (3)
C28'—Fe2—C23	176.3 (6)	N5—C20—C21	112.7 (3)
C21—Fe2—C23	68.94 (15)	N5—C20—H20A	109.1
C29'—Fe2—C22	175.1 (8)	C21—C20—H20A	109.1
C26—Fe2—C22	109.9 (5)	N5—C20—H20B	109.1
C27—Fe2—C22	125.1 (6)	C21—C20—H20B	109.1
C28'—Fe2—C22	141.2 (9)	H20A—C20—H20B	107.8
C21—Fe2—C22	40.83 (14)	C25—C21—C22	107.9 (3)
C23—Fe2—C22	40.91 (15)	C25—C21—C20	128.5 (4)
C29'—Fe2—C25	116.2 (8)	C22—C21—C20	123.6 (3)
C26—Fe2—C25	159.7 (7)	C25—C21—Fe2	69.9 (2)
C27—Fe2—C25	157.2 (7)	C22—C21—Fe2	69.6 (2)
C28'—Fe2—C25	114.6 (7)	C20—C21—Fe2	127.8 (3)
C21—Fe2—C25	40.58 (14)	C21—C22—C23	108.2 (4)
C23—Fe2—C25	68.70 (17)	C21—C22—Fe2	69.5 (2)
C22—Fe2—C25	68.37 (16)	C23—C22—Fe2	69.5 (2)
C29'—Fe2—C29	24.4 (7)	C21—C22—H22A	125.9
C26—Fe2—C29	67.8 (5)	C23—C22—H22A	125.9
C27—Fe2—C29	68.1 (7)	Fe2—C22—H22A	125.9
C28'—Fe2—C29	20.8 (7)	C24—C23—C22	107.5 (4)
C21—Fe2—C29	121.4 (4)	C24—C23—Fe2	69.9 (2)
C23—Fe2—C29	158.4 (5)	C22—C23—Fe2	69.6 (2)
C22—Fe2—C29	158.4 (5)	C24—C23—H23A	126.2
C25—Fe2—C29	105.9 (4)	C22—C23—H23A	126.2
C29'—Fe2—C24	113.9 (8)	Fe2—C23—H23A	126.2
C26—Fe2—C24	159.0 (7)	C23—C24—C25	108.4 (4)
C27—Fe2—C24	122.2 (7)	C23—C24—Fe2	69.5 (2)
C28'—Fe2—C24	140.9 (9)	C25—C24—Fe2	69.6 (2)
C21—Fe2—C24	68.48 (15)	C23—C24—H24A	125.8
C23—Fe2—C24	40.58 (17)	C25—C24—H24A	125.8
C22—Fe2—C24	68.27 (17)	Fe2—C24—H24A	125.8
C25—Fe2—C24	40.79 (14)	C21—C25—C24	108.0 (4)
C29—Fe2—C24	121.9 (4)	C21—C25—Fe2	69.6 (2)
C29'—Fe2—C28	56.4 (7)	C24—C25—Fe2	69.7 (2)
C26—Fe2—C28	40.8 (4)	C21—C25—H25A	126.0
C27—Fe2—C28	68.9 (7)	C24—C25—H25A	126.0
C28'—Fe2—C28	20.1 (7)	Fe2—C25—H25A	126.0
C21—Fe2—C28	107.0 (4)	C27—C26—C28	110.2 (12)
C23—Fe2—C28	159.7 (5)	C27—C26—Fe2	70.1 (9)
C22—Fe2—C28	123.2 (4)	C28—C26—Fe2	71.3 (7)
C25—Fe2—C28	122.0 (5)	C27—C26—H26A	124.9
C29—Fe2—C28	40.8 (3)	C28—C26—H26A	124.9
C24—Fe2—C28	158.2 (6)	Fe2—C26—H26A	124.9
C29'—Fe2—C30'	42.1 (10)	C26—C27—C30	109.5 (15)
C26—Fe2—C30'	51.6 (8)	C26—C27—Fe2	69.5 (9)
C27—Fe2—C30'	12.8 (10)	C30—C27—Fe2	71.8 (9)
C28'—Fe2—C30'	68.3 (11)	C26—C27—H27A	125.2
C21—Fe2—C30'	173.5 (10)	C30—C27—H27A	125.2
C23—Fe2—C30'	108.0 (10)	Fe2—C27—H27A	125.2
C22—Fe2—C30'	133.3 (10)	C26—C28—C29	105.3 (10)
C25—Fe2—C30'	144.5 (8)	C26—C28—Fe2	67.9 (7)
C29—Fe2—C30'	63.5 (9)	C29—C28—Fe2	69.2 (6)
C24—Fe2—C30'	113.3 (7)	C26—C28—H28A	127.3
C28—Fe2—C30'	73.8 (9)	C29—C28—H28A	127.3
N3—N1—N2	105.9 (3)	Fe2—C28—H28A	127.3
N1—N2—C7	106.2 (3)	C28—C29—C30	109.9 (11)
N1—N3—N4	114.0 (3)	C28—C29—Fe2	70.0 (6)
N1—N3—C6	122.9 (3)	C30—C29—Fe2	70.4 (6)
N4—N3—C6	123.1 (3)	C28—C29—H29A	125.0
N3—N4—C7	101.7 (3)	C30—C29—H29A	125.0
C19—N5—N8	108.9 (3)	Fe2—C29—H29A	125.0
C19—N5—C20	130.5 (3)	C27—C30—C29	105.0 (12)
N8—N5—C20	120.4 (3)	C27—C30—Fe2	67.6 (9)
C19—N6—N7	105.9 (3)	C29—C30—Fe2	68.4 (6)
N8—N7—N6	111.1 (3)	C27—C30—H30A	127.5
N7—N8—N5	106.3 (3)	C29—C30—H30A	127.5
C4—C1—C2	107.9 (4)	Fe2—C30—H30A	127.5
C4—C1—Fe1	69.8 (2)	C27'—C30'—C29'	106 (2)
C2—C1—Fe1	69.4 (2)	C27'—C30'—Fe2	72.2 (14)
C4—C1—H1B	126.1	C29'—C30'—Fe2	66.7 (13)
C2—C1—H1B	126.1	C27'—C30'—H30B	127.0
Fe1—C1—H1B	126.1	C29'—C30'—H30B	127.0
C1—C2—C3	107.7 (4)	Fe2—C30'—H30B	127.0
C1—C2—Fe1	70.5 (2)	C29'—C28'—C26'	109.1 (16)
C3—C2—Fe1	69.2 (2)	C29'—C28'—Fe2	69.5 (12)
C1—C2—H2B	126.1	C26'—C28'—Fe2	72.9 (11)
C3—C2—H2B	126.1	C29'—C28'—H28B	125.5
Fe1—C2—H2B	126.1	C26'—C28'—H28B	125.5
C5—C3—C2	107.0 (4)	Fe2—C28'—H28B	125.5
C5—C3—Fe1	69.9 (3)	C28'—C29'—C30'	108.7 (18)
C2—C3—Fe1	69.9 (3)	C28'—C29'—Fe2	71.0 (12)
C5—C3—H3A	126.5	C30'—C29'—Fe2	71.3 (14)
C2—C3—H3A	126.5	C28'—C29'—H29B	125.6
Fe1—C3—H3A	126.5	C30'—C29'—H29B	125.6
C5—C4—C1	108.5 (4)	Fe2—C29'—H29B	125.6
C5—C4—Fe1	69.6 (2)	C30'—C27'—C26'	111 (2)
C1—C4—Fe1	70.2 (2)	C30'—C27'—Fe2	69.2 (14)
C5—C4—H4B	125.8	C26'—C27'—Fe2	70.5 (11)
C1—C4—H4B	125.8	C30'—C27'—H27B	124.7
Fe1—C4—H4B	125.8	C26'—C27'—H27B	124.7
C4—C5—C3	108.9 (4)	Fe2—C27'—H27B	124.7
C4—C5—Fe1	70.8 (3)	C27'—C26'—C28'	105.6 (16)
C3—C5—Fe1	69.6 (3)	C27'—C26'—Fe2	70.2 (11)
C4—C5—H5A	125.6	C28'—C26'—Fe2	66.4 (11)
C3—C5—H5A	125.6	C27'—C26'—H26B	127.2
Fe1—C5—H5A	125.6	C28'—C26'—H26B	127.2
N3—C6—C11	111.9 (3)	Fe2—C26'—H26B	127.2
N3—C6—H6A	109.2		

### Hydrogen-bond geometry (Å, °)

**Table:**

*D*—H···*A*	*D*—H	H···*A*	*D*···*A*	*D*—H···*A*
C20—H20B···N2^i^	0.97	2.49	3.391 (5)	154

Symmetry codes: (i) −*x*, −*y*+1, −*z*+1.
